# Bionic Design for Reducing Adhesive Resistance of the Ridger Inspired by a Boar's Head

**DOI:** 10.1155/2017/8315972

**Published:** 2017-07-03

**Authors:** Jianqiao Li, Yunpeng Yan, Benard Chirende, Xuejiao Wu, Zhaoliang Wang, Meng Zou

**Affiliations:** ^1^Key Laboratory for Bionics Engineering of Education Ministry, Jilin University, Changchun 130022, China; ^2^University of Mpumalanga, Private Bag X11283, Mbombela 1200, South Africa

## Abstract

The main feature of the boar's head used to root around for food is the front part, which is similar to the ridger in terms of function, load, and environment. In this paper, the boar's head was selected as the biological prototype for developing a new ridger. The point cloud of the head was captured by a 3D scanner, and then, the head surface was reconstructed using 3D coordinates. The characteristic curves of the front part of the boar's head were extracted, and then, five cross-sectional curves and one vertical section curve were fitted. Based on the fitted curves, five kinds of bionic ridgers were designed. The penetrating resistances of the bionic ridgers and traditional ridger were tested at different speeds in an indoor soil bin. The test results showed that bionic ridger B had the best penetrating resistance reduction ratio of 16.67% at 4.2 km/h velocity. In order to further evaluate the performance of the best bionic ridger (bionic ridger B), both the bionic ridger and traditional ridger were tested in a field under the same working conditions. The field results indicate that the bionic ridger reduces penetrating resistance by 6.91% compared to the traditional ridger, and the test results validate that the bionic ridger has an effect on reducing penetrating resistance.

## 1. Introduction

Tillage is an agricultural land preparation process by mechanical means such as digging, stirring, and overturning the soil [1]. Tillage examples include ploughing, rototilling, rolling with cultipackers or other rollers, harrowing, ridging, and cultivating with cultivator shanks [2]. It is negatively affected by soil adhesion which exists widely in all kinds of tillage machines during wet conditions. Soil adhesion increases ridging resistance and power consumption by more than 30% and 30%~50%, respectively, and decreases seed emergence rate of seeding machines by 5%~10% [3, 4]. Soil adhesion is therefore regarded as a significant problem affecting field performance of tillage machinery. Scholars are therefore devoted to the study of the theory and mechanism of reducing soil adhesion effectively and further explore the methods of penetrating resistance [5].

In order to reduce the energy consumption generated by tillage resistance [6], several methods of reducing soil-tool adhesion have been reported, such as heat treatment, lubrication between the tool and soil, use of different materials and coatings, vibrations, electromagnetic field applications [7], optimization of the tool geometry and operational conditions [8–10], designing of a reversible plough or ridger [11], and design modifications of a bionic surface [12].

Soil animals have a better function of reducing adhesion due to their long evolution period and can move freely with very little soil adhesion even under wet soil conditions [12, 13]. By means of bionics, scholars can study all kinds of biological prototypes with the function of reducing adhesion and resistance in nature, conduct bionic research, and explore the excellent functions of reducing adhesion [3]. This possesses important practical significance and broad application prospects in reducing adhesion of agricultural machinery.

A ridger is one of the most important soil-engaging components on agricultural implements such as a combined tillage machine, soil preparation ridging machine, and fertilizer applicator, applied widespreadly in both primary and secondary tillages [14, 15]. This paper takes a ridger as the study object leading to the designing of a low-resistance ridger according to principles of bionics. The biological prototype (boar's head) is scanned, and its feature parameters are extracted so that characteristic curves are produced for the purpose of designing and manufacturing bionic ridgers. These bionic and traditional ridgers are first tested in a soil bin and then further evaluated under actual field conditions.

## 2. Biological Prototype

### 2.1. Prototype Selection

The wild boar has a bulky, massive body with short and relatively thin legs. The trunk is short and heavy, and the hindquarters are comparatively underdeveloped. The region behind the shoulder blades rises into a hump, and the neck is short and thick, to the point of being nearly immobile. The head is very large, taking up one-third of the body's entire length. The structure of the head is well suited for digging as it roots around for food. The head acts as a plough or ridger, while the powerful neck muscles allow the animal to upturn considerable amount of soil: it is capable of digging 8–10 cm into frozen ground and can upturn rocks weighing 40–50 kg [16].

The boar has a behavior of arching for a long time as it looks for food in the soil, and this requires the excellent function of reducing resistance when the snout contacts the soil. This function was developed over a long time of natural evolution and optimization. Therefore, the boar provides an excellent bionic prototype for the study of reducing adhesion and resistance of the ridger. A wild boar was selected and its head was scanned and its main features for reducing resistance were analyzed.

**Figure 1:**
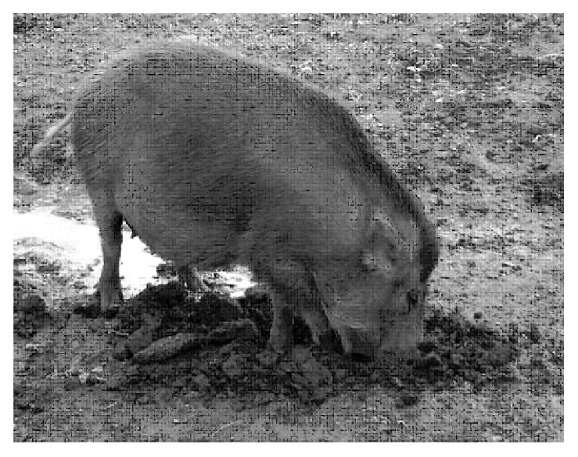
The boar arching for food in the soil.

The sample was gotten from Changbai Mountain wild animal domesticating location in Baishan City, Jilin Province, P.R. China. The picture of the boar is shown in Figure 1.

### 2.2. 3D Scanning of the Wild Boar Head

**Figure 2:**
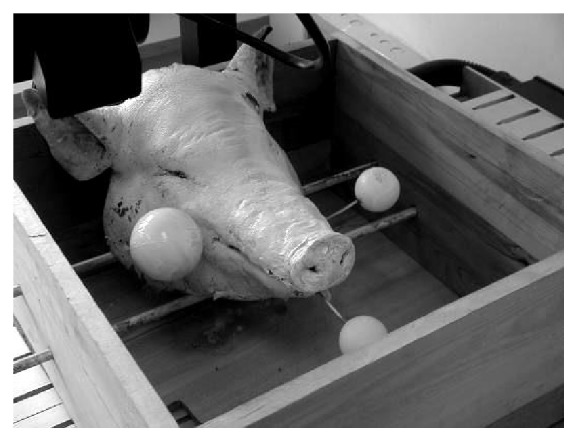
The boar sample after the coloring process.

**Figure 3:**
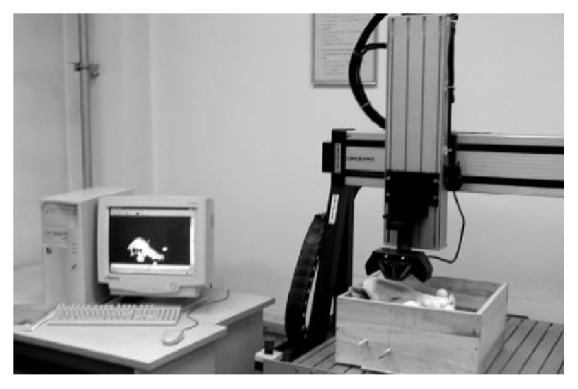
Collection of the cloud data from the boar head.

The geometrical characteristics of 3D point cloud data of the boar's head were created and analyzed using a nontouch laser 3D scanner. The 3D point cloud data could only be obtained by collecting the reflected light from the surface, but the boar's light surface made the process difficult. Therefore, the sample was lacquered in white in order to enhance scanning quality.  Figure 2 is the boar head sample after the coating process. The cloud data collection set-up is shown in Figure 3.

The boar's head is a complex geometrical object with a diversified and irregular appearance, so it is difficult to get the whole data in one set-up. To solve this problem, various angles, repeated scanning methods, and three-point fixing were adopted to keep the position of datum accurate.

Every point cloud obtained by scanning on a laser scanner included lots of noises from sources such as stent, shim, and table. These noises should be removed before the treatment of points, lines, and surfaces. A tiny burr which was formed around the fitting sphere due to light scattering by the boar head sample also had to be deleted.

### 2.3. 3D Model Reconstruction

**Figure 4:**
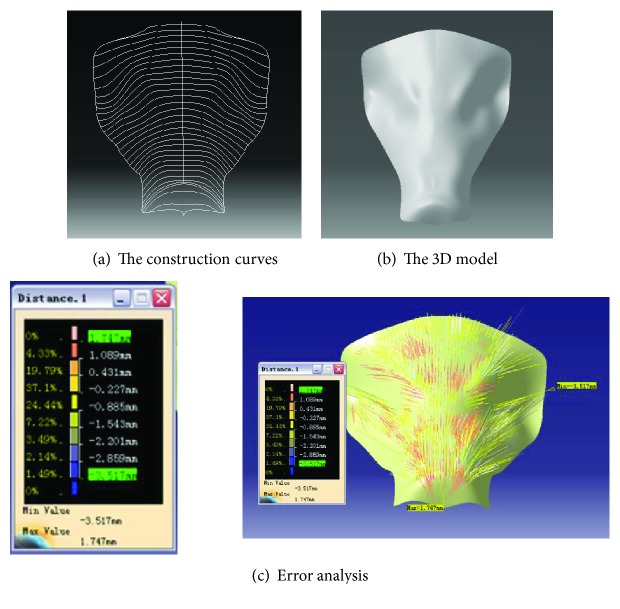
3D model reconstruction of the boar head.

In this 3D model reconstruction method, the point clouds generate curves which in turn generate the surface. This is in consideration of the complexity of the geometric properties of the boar's head. Firstly, the point clouds were divided, and secondly, the point clouds intersected a set of parallel planes to obtain curves. Thirdly, the surface was generated from the curves, as shown in Figures 4(a) and 4(b).

Some tiny biological characteristics on the head such as small tuber and scallop were easily smoothened automatically by software resulting in errors between the reconstructed surface and original point cloud, and these errors had to be analyzed.

The distance between the points of the 3D model and the original point cloud was used to analyze the error displayed by color band, and the percentage of each distance was shown simultaneously. As shown in Figure 4(c), the positive error is 1.747 mm and the negative one is −3.517 mm. The negative error is near the eyes of the sample. The eyes are concave in the original sample, but the point cloud does not completely reproduce it by scanning. The positive error appears at the top of the snout where the curvature changes greatly. This error also arises from smoothening during reconstruction. The range of errors is around 1/100 compared with the volume of the sample which is 300 mm × 30 mm × 300 mm. The error of the surface met the engineering design standards after analyzing the distance between geometric modeling and the original point cloud and Gaussian curvature and mapping.

## 3. Design and Preparation of the New Ridge

### 3.1. Feature Parameter Extraction

**Figure 5:**
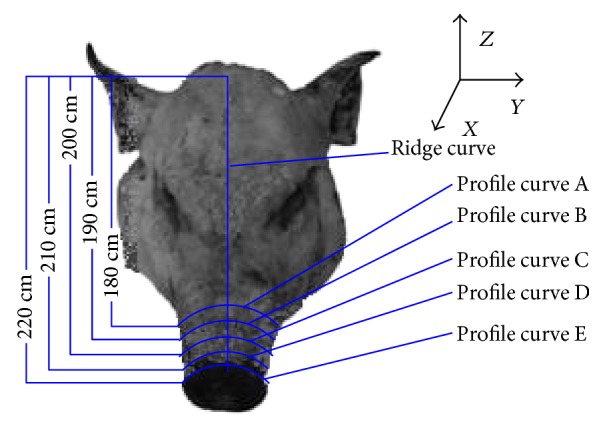
Extraction and analysis of profile curves.

**Figure 6:**
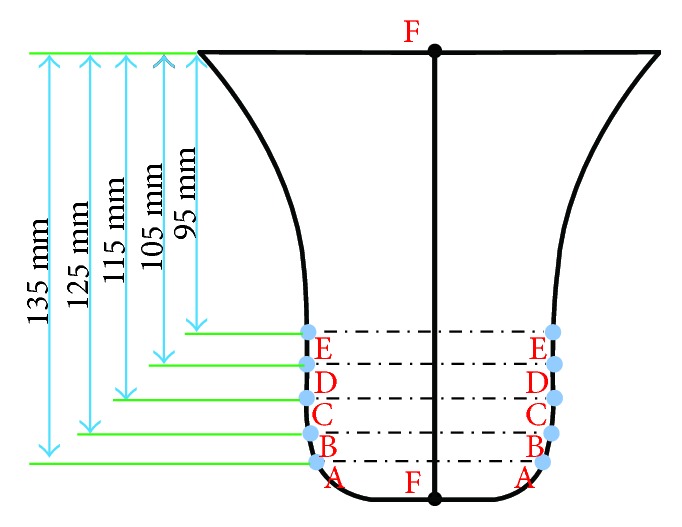
The extracting position of characteristic curves and the related plane of the ridge curve.

It is important to analyze the surface of the boar's head, as it plays a significant role in rooting around for food. In the design of the shovel part of the ridger, six curves were chosen to analyze the geometric characteristics corresponding to the soil-engaging component. The XY direction plane is regarded as the base plane, which is at the base line of the model in CATIA software. Five planes parallel to the base plane intersected the ridge curve at 180 mm, 190 mm, 200 mm, 210 mm, and 220 mm, respectively, from the base plane, producing 5 curves as shown in Figure 5. These 5 curves are referred to as profile curves, numbered A, B, C, D, and E, respectively, and the interval of intersection is 10 mm. The uppermost curve in the lateral view of the boar's snout surface is named the ridge curve as also shown in Figure 5.  Figure 6 shows the extracting position of 5 profile curves and the ridge curve on the boar's snout.

**Figure 7:**
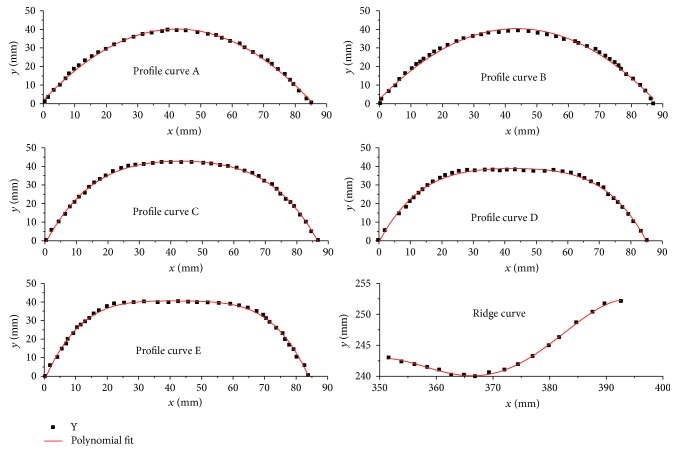
Fitting equations of the profile curves and ridge curve.

The fitting equations obtained from profile curves A to E and the ridge curve are shown in Figure 7, and their point coordinates are extracted from these equations. The fitting equations of curves A–F are best described by (1), and its coefficients are shown in Table 1.

(1) $$\begin{array}{c} y=\alpha x+\beta x^{2}+\chi x^{3}+\eta . \end{array}$$

**Figure 8:**
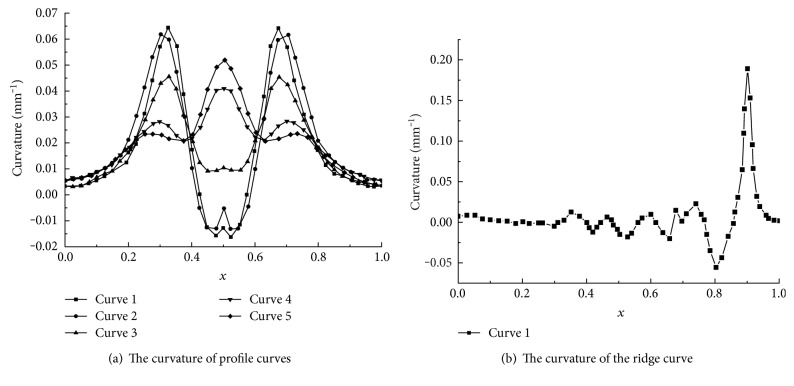
The curve analysis of the profile and ridge curves.

The curvatures of the 5 profile curves and ridge curve were analyzed in Figures 8(a) and 8(b), respectively. The extracted coordinates are used to plot the relative curvature distribution graph. The total length of the *x*-axis is 1, and the *y*-axis shows the profile and ridge curvatures. The positive direction of the *x*-axis is from the top to bottom of curves A to E. All the profile curves are drawn on the same XY plane so as to allow relative comparison of the overall profile curvature.

For all the profile curves 1 to 5, the curvatures change acutely at the following regions: *x* = 0.1 to 0.3, 0.2 to 0.5, and 0.5 to 0.8 as shown in Figure 8(a). The corresponding part has the function of separating soil when the boar arches to the earth and has an advantage of reducing resistance.

The curvature of the ridge curve is shown in Figure 8(b). Point density is defined as 200 because of the longer ridge curve compared to 5 profile curves. The total length of the *x*-axis is 1, and the *y*-axis shows the curvature. The positive direction of the *x*-axis is from the left to right.

The curvature of the ridge curve changes greatly from 0.8 to 1 along the *x*-axis as shown in Figure 8(b). This region is corresponding to the soil-engaging part—the snout. It is clear that the geometrical shape changes drastically and is adapted to soil resistance reduction.

The analysis on these 6 curves makes instructional sense for the research on the bionic ridger and is also the basis for deducing the bionic design parameters. As shown in Figure 8(a), the curvature extreme values of profile curves A to E are very small, and hence, the curvature changes are also tiny. It means that the profile curves are relatively smooth. The maximum curvature of the ridge curve appears on the snout, but the ranges are also tiny on other sections; hence, the transition of curves is smooth.

### 3.2. Bionic Design of the Ridger

**Figure 9:**
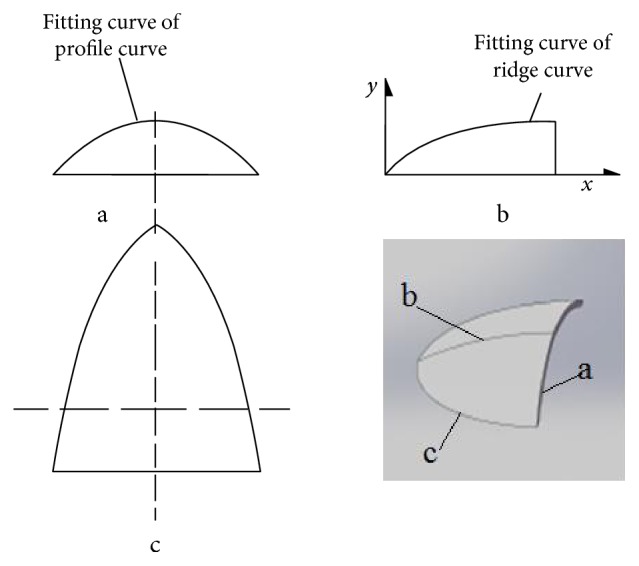
The design drawing of the bionic ridger.

The surface of the bionic ridgers is generated using software, according to the fitting equations of the profile curves and ridge curve obtained from the bionic prototype analysis. Considering that the curvature of the ridge curve equation reflects concavity, it can easily pile the soil on the front of the ridger and will not help in dispersing the soil and reducing resistance. Therefore, the ridge curve equation should be applied in reverse, such that the shovel has a convex curvature to facilitate the soil dispersion. The design drawing of the bionic ridger is shown in Figure 9, and Figures 9, a, 9, b, and 9, c are orthographic views based on the profile and ridge curves.

The biological geometric characteristics of the snout were applied to the bionic ridger design, and the bionic ridger possesses those characteristics which can help in reducing resistance. In order to verify if these characteristics had an effect on reducing resistance, both the bionic ridger and traditional ridger had the same design specifications except for the difference in shovel configuration. According to the measured dimensions of the traditional ridger, the width of the shovel is 150 mm. The height and length of the shovel are 35 mm and 170 mm, respectively, when it is horizontal. Consequently, the designed bionic ridger had width, height, and length of 150 mm, 35 mm, and 170 mm, respectively.

**Figure 10:**
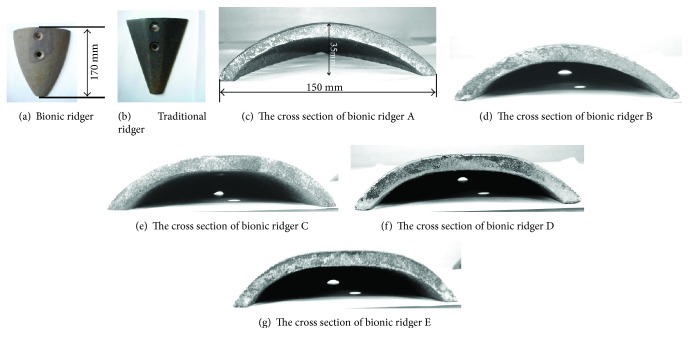
The bionic ridgers and traditional ridger.

Five different kinds of bionic surfaces were generated using the 5 profile curves and ridge curve. Five different kinds of shovels were developed by properly machining the surfaces according to the profile curves. The designed bionic ridgers were analogous, because of the similar fitting surface and the processing technique. The bionic ridgers generated by curves A, B, C, D, and E were marked as bionic ridgers A, B, C, D, and E, respectively. The bionic and traditional ridgers are shown in Figures 10(a) and 10(b), respectively, and the cross-sectional diagrams of these five bionic ridgers are shown in Figures 10(c), 10(d), 10(e), 10(f), and 10(g).

**Figure 11:**
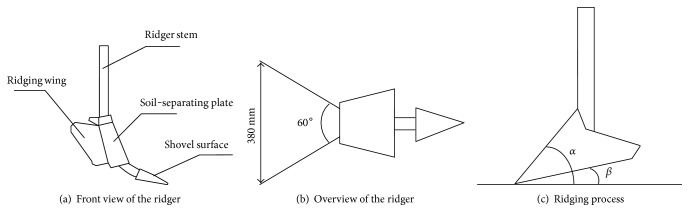
Schematic drawing of the ridger.

The project mainly focused on whether the bionic ridger with snout geometrical characteristics could reduce resistance, so other parameters were determined by empirical means. These are wing open angle, wing width, penetration angle, and penetration clearance angle set at 60°, 380 mm, 25°, and 10°, respectively, as shown in Figures 11(a), 11(b), and 11(c).  Figure 11(a) is the front view of the ridger and shows the main parts. The overview in Figure 11(b) shows the wing open angle and wing width.  Figure 11(c) is the ridging process, and *α* and *β* are the penetration angle and penetration clearance angle, respectively.

## 4. Experimental Verification

### 4.1. Indoor Soil Bin Test and Results

In order to find the shovel with the greatest resistance reduction effect through comparing the penetrating resistance between bionic ridgers designed using snout characteristics curves and the traditional ridger under the same conditions, the indoor soil bin tests were carried out at Jilin University, China. The soil used in the indoor test is yellow clay in the northeast region of China, whose moisture content and hardness are 20.8% and 74 N/cm^2^, respectively. A BLR-1260 tension-compression sensor was used in these tests, and the nonlinear error of this sensor is ±0.5% FS.

One bionic ridger with the best resistance reduction effect was selected from the 5 kinds of bionic ridgers by processing test data and comparing the penetrating resistance between the traditional ridger and bionic ridgers. Tests were conducted at 5 different speeds (1.0, 1.8, 2.6, 3.4, and 4.2 km/h) and 12 cm ridging depth. The penetrating resistance of every ridger was retested three times at the same speed and then averaged as penetrating resistance at a particular speed. The soil moisture content and soil hardness were kept as much consistent as possible during every process of penetration. The soil moisture content was tested every day, and the soil was spread after each test. It was also overturned using a rotary tiller and compacted by rollers after the test every day. In order to acquire stable reliable results, the effective length covered during the indoor soil bin test is 20 m.

**Figure 12:**
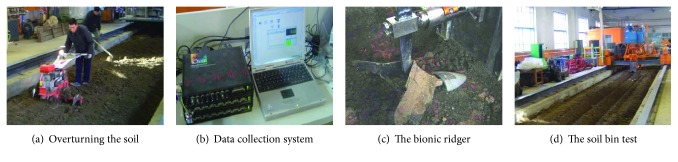
Indoor soil bin test.

These tests of penetrating resistance were conducted in the indoor soil bin in Key Laboratory of Bionics Engineering of Education Ministry, Jilin University. After three times of ridger penetration into the soil, the soil was turned and compacted so as to ensure that the test is conducted under the same soil conditions. As shown in Figure 12(a), a worker is overturning the soil in the soil bin.  Figure 12(b) shows the data acquisition system, and the black instrument on the left is a virtual instrument, which is connected with a tension-compression sensor. The test data is displayed on the computer after internal data collection and processing.  Figure 12(c) shows the bionic ridger before penetration and Figure 12(d) shows the bionic ridger during the penetration process. The shovel is the only part changed in the test.

As shown in Figure 3(), the penetrating resistances of 5 kinds of bionic ridgers and one traditional ridger at 5 different speeds are plotted by processing the collected data. Figure 3() shows that the bars of bionic ridgers A, B, and C at 5 different speeds are all below the bar of traditional ridger F. It shows that the penetrating resistances of bionic ridgers A, B, and C are all lower than that of traditional ridger F and bionic ridger B has the least overall penetrating resistance. All of the penetrating resistances have the tendency to increase with an increase in the travelling speed. During the test, the least resistance is 0.1558 kN, which occurs on bionic ridger C at a speed of 1 km/h. The greatest resistance is 0.2378 kN, which occurs on bionic ridger E at a speed of 4.2 km/h.

The resistance reduction efficiency of the ridger is equal to the difference in the value of the penetrating resistances between traditional ridger F and the bionic ridger divided by the penetrating resistance of traditional ridger F and multiplied by 100. Bionic ridger B has the best average resistance reduction efficiency of 13.66%, and its maximum is 16.67% which occurs at the speed of 4.2 km/h. Bionic ridgers A and C reduce resistance by an average of 7.46% and 9.73%, respectively, while bionic ridgers D and E actually increase resistance. As we can see from the analysis, the geometric characteristics of the bionic ridger which has the effect on reducing resistance were based on the profile and ridge curves of the boar's snout. This provides a research direction for reducing resistance by a bionic ridger.

### 4.2. Field Experiment and Result

The aforementioned indoor soil bin test shows that bionic ridger B has the best effect on reducing resistance; however, it had to be further verified by a field experiment. In order to validate the effect on reducing resistance, a contrast test was adopted. The traditional ridger and bionic ridgers were installed on the prototypical original and Jilin University-developed machine, respectively, and penetrating resistance was tested at 60-horsepower tractor's two gear ratios. It is basically arranging 2 groups of contrast test that is adopting the traditional ridger and bionic ridgers on the original machine to measure penetrating resistance at 2 gear ratios. Then, the same process is done on the Jilin University-developed machine. The bionic ridger used in the test was bionic ridger B which had the best effect on reducing resistance during the indoor soil bin test, and the field tests were done at a depth of 120 mm. The soil was the same as the indoor soil, and its moisture content and hardness were 20.28% and 81.71 N/cm^2^, respectively. Two gear ratios were applied to the tractor. The test is shown in Figure 14(a), and the effect after penetration by the ridger is shown in Figure 14(b).

The penetrating resistance results of the traditional ridger and bionic ridgers are summarized, and the resistance reduction efficiency of bionic ridgers is calculated for all the conditions. The resistance reduction efficiency is calculated using

(2) $$\begin{array}{c} \delta =\frac{F_{t}-F_{b}}{F_{t}}, \end{array}$$

where *F*_t_ is the resistance of the traditional ridger and *F*_b_ is the resistance of the bionic ridger.

**Figure 13:**
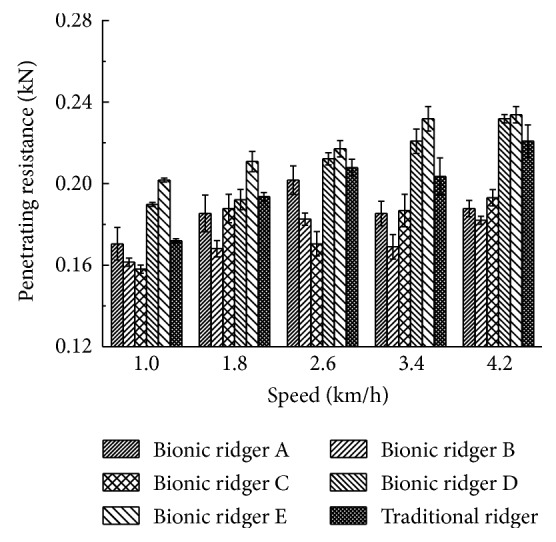
Resistance of the bionic ridger and traditional ridger.

**Figure 14:**
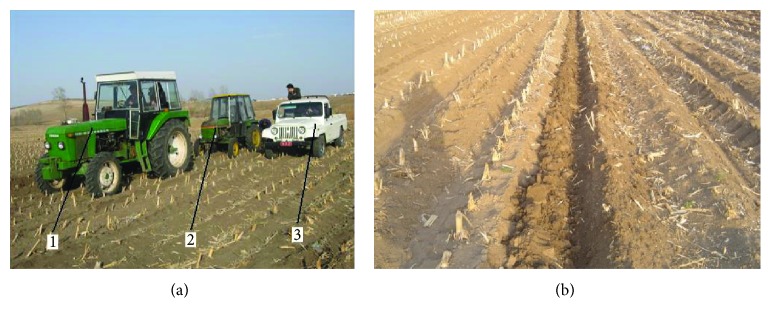
Field experiment. (a) 1 is the main pulling tractor, 2 is the experiment tractor with the ridger, and 3 is the measurement device. (b) The effect of penetration by the ridger.

**Figure 15:**
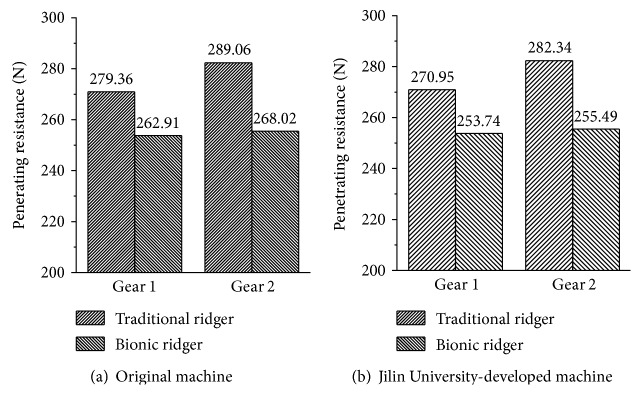
The comparison table of penetrating resistance between the bionic ridger and traditional ridger.

As can be seen in Figure 13, the penetrating resistances of the bionic ridger on the original machine are reduced by 16.45 N and 21.04 N, respectively, compared to those of the traditional ridger for the 2 gear ratios. The resistance reduction efficiencies are 5.89% and 7.28%, respectively, and the average is 6.58%. On the Jilin University-developed machine, the penetrating resistances of bionic ridgers reduce by 17.21 N and 26.85 N compared to those of the traditional ridger, respectively. The resistance reduction efficiencies are 6.35% and 9.51%, respectively, and the average is 7.93%. On both the Jilin University-developed machine and original machine, the penetrating resistances of bionic ridgers are less than that of the traditional ridger (see Figures 15(a) and 15(b)); bionic ridgers reduced resistance by 6.91% compared to the traditional ridger, and it validates that bionic ridger B had an effect on reducing resistance.

### 4.3. Discussion

The traditional methods of reducing adhesion and resistance are divided into the following kinds: aeration or liquid-filled method, thermal desorption method, vibration method, electroosmotic method, mechanical method, and surface modification method. Experiment indicates that the average resistance is reduced by 16% by injecting polymer solution in a concentration of 3% into the upper surface of the plow board. The resistances are reduced by 8%–12% and 2.5%–3.5% in water culture and in dry farming, respectively, through building “comet through hole” on the plow's surface. The plow named “bulge 20” is developed by changing the material, the size and dimensions of bulges, and the body surface of the plow, and resistance is reduced by 15%–18% compared with that of the homogenous plow. And the resistance of the bionic ridge used in this experiment is reduced by 16.67%.

It is quite difficult to study reducing adhesion and resistance because of the variety of soil touching parts of a terrain machine, variability of sticky soil, complexity of adhesion interface, and randomness of an adhesion process. A classical theory and traditional method are limited by many factors, so we have to seek solutions in an up-to-date thought. Under the biological principles named the survival of the fittest, the creatures in nature possess the function of desorption and drag reduction during a long-drawn evolution. The adhesion and resistance in a terrain machine are solved by learning self-adaption of soil animals. In this paper, characteristic curves on the wild boar are extracted to design ridgers and ridging resistance is reduced effectively. However, it is difficult to manufacture bionic ridgers and it cannot loosen the soil very well.

## 5. Conclusions

1. The geometrical characteristics of 3D point cloud data of the boar's head were created and analyzed by a nontouch laser 3D scanner. After 3D model reconstruction, the maximum errors on positive direction and negative direction were 1.747 mm and −3.517 mm, respectively, and the range of errors is around 1/100 compared with the volume of the sample which is 300 mm × 30 mm × 300 mm.
2. Five profile curves and the ridge curve were chosen as characteristic curves corresponding to the soil-engaging section on the boar's head, and six fitting curves were obtained. Five kinds of bionic ridgers were then designed according to the six characteristic curves at the soil-engaging section of the boar's head.
3. The soil bin tests show that bionic ridgers have an effect on reducing resistance, and bionic ridger B has the highest average resistance reduction efficiency, which is 13.66%. The bionic ridgers A and C reduce resistances by 7.46% and 9.73%, respectively. The resistance reduction efficiency of bionic ridger B is 16.67% when the travelling speed is 4.2 km/h.
4. The field experiment shows that the penetrating resistance of bionic ridgers is less than that of the traditional ridger. Overall, bionic ridgers reduce resistance by 6.91% compared to the traditional ridger.

## Figures and Tables

**Table 1:** Coefficients of fitting equations of curves A–F.

Coefficient curve	*α*	*β*	*χ*	*η*
A	1.81	−0.02	0	1.89
B	1.74	−0.02	0	2.52
C	3.20	−0.03	0.001	1.54
D	3.13	−0.097	0.001	−0.39
E	3.39	−0.133	0.002	−2.21
F	6429	−26.11	0.004	−592810
